# The work of a children’s psychiatric clinic in the context of the COVID-19 pandemic in russia

**DOI:** 10.1192/j.eurpsy.2021.570

**Published:** 2021-08-13

**Authors:** D. Dovbysh, M. Bebchuk, Y. Zhorina, E. Gimranova, S. Timoshenko, E. Popil

**Affiliations:** 1 Pedagogy And Medical Psychology, Federal State Autonomous Educational Institution of Higher Education I.M. Sechenov First Moscow State Medical University of the Ministry of Health of the Russian Federation (Sechenov University), Moscow, Russian Federation; 2 Child And Adolescent Psychiatry, Scientific-practical Children’s and Adolescents Mental Health Center n.a. G. Sukhareva, Moscow Department of Health Care, Moscow, Russian Federation

**Keywords:** Child Psychiatry, COVID-19, Patient Care Team, Health Personnel

## Abstract

**Introduction:**

In the context of the Covid-19 pandemic, healthcare workers experienced significant distress. At the same time, concern for the safety and well-being of employees remained important priorities to ensure the quality of care for children with mental illness.

**Objectives:**

To study the specifics of the experience of the Covid-19 pandemic among employees of a children’s psychiatric clinic, highlight the existing among them attitudes about the pandemic and form administrative decisions to improve the quality of care for children.

**Methods:**

380 employees voluntarily took part in the study (group 1 (G1): 115 people who worked directly with Covid-19 and group 2 (G2): 265 people without this experience) from 05/18/2020 to 05/20/2020. The author’s questionnaire included the following blocks: 1) attitude towards patients and colleagues; 2) emotional experiences; 3) ways of coping; 4) social support; 4) finance.

**Results:**

The main motive when deciding to work with Covid-19 was the motive of professional duty (25.4% of participants). There are a number of significant differences between group 1 and group 2: participants in G1 are characterized by denial of special experiences associated with Covid-19, seeking help from colleagues in difficult working conditions, reliance on family members and a positive vision of administrative decisions significantly more than participants G2. Relatives of G1 participants are less concerned about their future and health.

**Conclusions:**

The personnel decisions made on the basis of the research allowed the clinic’s team to provide quality care to children and families throughout the pandemic.

